# Osteogenesis and Osteoplasty in Crushing Lesions of the Extremities1Read at the Second Annual Meeting of the New York State Association of Railway Surgeons, New York Academy of Medicine, November 14, 1892.

**Published:** 1892-12

**Authors:** Thomas H. Manley

**Affiliations:** 302 W. Fifty-third Street; New York


### Buffalo Medical and Surgical Journal

Vol. XXXII. DECEMBER, 1892. No. 5.

### Original Communications.

### OSTEOGENESIS AND OSTEOPLASTY IN CRUSHING LESIONS OF THE EXTREMITIES.[1]

By THOMAS H. MANLEY, M. D., New York.

The study of lesions of the extremities arising in consequence of great disorganizing force, which involve the osseous and arthritic elements, is one, alike, interesting and profitable to surgeons who are largely concerned in dealing with traumatism, particularly such as are employed by railroad companies, or reside in great commercial, industrial, or manufacturing centers. While the methodical pursuance of this subject is particularly desirable to active surgeons, yet every practitioner so placed that he may be called on to deal with injuries of the bones and the overlying soft parts, in order to secure the best results, should not neglect it.

In our time, since the rapid and extensive expansion of the surgery of the abdomen and other serous cavities, the management of fractures and dislocations, simple and complicated, seems to have received less attention than its importance deserves. Modern investigation, combined with improved mechanical facilities, the gradual but steady diminution in number of the old masters, with the advent of a large number of young recruits filled with a laudable ambition to reach the summit of fame, have each and all conspired to revolutionize the principles of surgery as taught within the recollection of many living. We have known venesection when it was regarded as one of the most valuable of prophylactics in every serious injury when the supervention of inflammation was anticipated. This had its day ; then it was condemned, and it was alleged that " the lance killed more than the sword." Mercury,

1. Read at the Second Annual Meeting of The New York State Association of Railway Surgeons, New York Academy of Medicine, November 14, 1892.

internally administered, was considered a sovereign remedy, used conjointly with emetics, diaphoretics, and purgatives, at the onset of an inflammation, which had its origin in violence. But homeopathy took the field, and though Hahnemannism was not unconditionally accepted, it cannot be denied that it struck a crippling blow at the prevailing therapeutic theories of the time. Then we had wet dressings, again we had dry; at one time cold, and other wetted. At length Listerism, or antisepticism, took a vantage place, and the doctrine was pretty generally accepted by the civilized world. Finally, it did seem, for a time, that the millennium had come. With no more pain, with no danger of consecutive inflammation, tetanus erysipelas, or gangrene, simple or compound fractures, compound dislocations, resections or amputations must no longer give us serious concern. But, alas, for the fallibility of all things human, orthodox, antiseptics belongs to the history of the past; for we have at last realized what should have been self-evident to us all when this seductive theory was first thrust upon us, viz., that everything worthy of the name is an irritant and a foreign substance. Time, and a very short time, too, demonstrated that the most useful of all so-called antiseptics was mercury, applied externally, in a liquid state, by which it might be most readily assimilated.

More than a year ago, I called the attention of the profession to the lethal action of antiseptics and hot water on bare, bleeding, denuded bone, and reported cases in which, though primary union was present and complete in amputations, useless, painful stumps have often resulted from an insidious osteomyelitis, requiring a further sacrifice of the limb. The antiseptic wave swept over. It is already practically lost in the hazy oblivion of the past, and asepsis has followed in its wake. The latter term would imply that good surgery in the past did not always include the most rigorous cleanliness, which is wholly without foundation.

With those shifting, vacillating theories and doctrines, which have each come and gone in the present century, we must needs be not a little confused in deciding which to adopt, or which to discard. The fact is, that each has its application, and that no one of them can be indiscriminately employed in every case. Careful study, combined with ample observation of cases, will enable us to better comprehend the cases in which either asepsis or antisepsis may be most appropriately employed.

DISORGANIZATION OF THE OSSEOUS STRUCTURES OF THE UPPER OR LOWER EXTREMITY, RESULTING FROM RAILROAD ACCIDENT OR PHYSICAL VIOLENCE.

In order to more fully comprehend the consequences arising from a railroad accident, in which the shafts of bones or the articulations have suffered injury or damage to their component elements, we must first draw a line of distinction between the quality of motors employed on railroads, and the carriages for passengers; the shape of the rail, or wheel, and weight or velocity of the vehicle in motion. In our thrifty American cities and villages, with the general prosperity of our people, and facilities for cheap, rapid, and pleasant travel, the steam, electric, and horse tram have come into very general use, and hence, with us, millions ride daily who formerly walked.

We can readily understand that there must be a wide difference in the quality of an injury inflicted by the swift movement of a limited flyer of tremendous weight and the velocity of the wind, and that occasioned by the lighter, slower-moving street car. Certainly, since tramway companies have utilized steam in the cable movement for direct traction; and since electricity is so rapidly displacing horses, this sort of travel is becoming proportionally more dangerous in the event of collision or derailment. As accidents and injuries from tramway travel, though not possessing the attributes of destruction peculiar to steam railways occupied by passenger and freight travel, they are, by all odds, the most frequent, and it would seem that a consideration of them should be included in this subject.

The sacrifice of a limb in tramway travel is a comparatively unusual event, while the reverse is the case in crushing injuries of the extremities on railroads. In the one, we may concern ourselves chiefly with the local lesion; in the other, this may be but a minor consideration compared to the contemporaneous, by bodily shock, laceration of an internal organ, concussion, or internal hemorrhage.

It is quite impossible to discuss the osseous lesions of the extremities without considering the soft parts, particularly the muscular, neural, and vascular structures. The shafts of bones acted upon by muscular levers, animated by nerve filaments, and nourished by vessels in their undisturbed contiguity, these latter are essentially constituent parts of one and the same organ in health, so that when maimed or damaged by accident, that intimate relationship cannot be disturbed with impunity. Accordingly, in this instance, while the osseous structures will chiefly occupy us, the adjacent structures will not be lost sight of. In tramway accidents, we most often encounter damage to the lower extremities, while in railroad casualties proper, injuries to the hand and forearm predominate in number. With the former, the misadventure occurs most frequently while the passenger is mounting or leaving the car while in motion, the trunk being projected outward and the feet inward. Besides, the slow movement of the street car permits one, in the flitting second intervening before the rolling wheel strikes, to instinctively save his head and arms. With heavy rolling stock, the coupling of cars causes many extensive contusions of the arms, hands, and fingers.

In collision or derailment, the suddenness is often so great that the instinct of self-preservation is powerless to act. Very much information can be gathered in making our examination, before we inspect the injured, in street railway accidents, by a methodically conducted interrogatory: First, was the car moving at a rapid rate of speed? Second, was it heavily loaded with passengers? Third, did one or two wheels pass over the limb? Fourth, was the injured struck by the horses, knocked down, trodden on, and then run over by one or more wheels?

We now come to an inspection of the patient; to ascertain his general condition, especially with reference to his cerebro-spinal system, his internal organs, and the state of the circulation. If the patient be a child or growing youth, we will always find collapse and shock much more marked than with the same extent of injury in the healthy adult.

Having regarded his general condition, we approach the examination of the injured parts, for the consideration of the management of which, in lesions of the extremities, the present study is undertaken.

### LESIONS OF THE EXTREMITIES INDUCED BY STREET CAR INJURIES.

This class of injuries are quite peculiar to themselves, inasmuch as the danger inflicted is generally of a local character, and there is seldom marked collapse or shock present, unless a large blood vessel has been opened.

### CLASSIFICATION OF THESE CASES.

(1.) Those consisting of contusion or laceration of the soft parts. (2.) The above, associated with fracture, simple, multiple, compound, or comminuted. (3.) Dislocation, or fracture-dislocation. (4.) Loss of substance, partial or complete traumatic amputation.

I.

Traumatic lesion of the soft parts, whether penetrating or not, when not of an extensive area, and not attended with laceration of important nerve trunks or blood vessels, and do not implicate joints in the healthy individual, commonly pursue a prompt course toward recovery, under appropriate treatment. They become dangerous to life, or may compromise the ultimate full functional use of the limb only by the development of paralysis, erysipelas, gangrene, arthritic, or muscular anchylosis.

II.

The mangling of a limb, in which all its elements share in the consequences of vulnerant force, demand a full knowledge of the recuperative powers of the economy, sound judgment, skill, and experience in its management.

Ollier, surgeon to the Hotel Dieu, of Lyons, has recently published an unrivaled work on Osteoplastic Operations, a translation of which into English will soon be published by a well-known Western surgeon. Ollier's work has gone a long way in the direction of extreme conservatism in the management of that class of injuries which, heretofore, were only too often promptly dealt with by the saw and amputating knife. And here I beg to acknowledge a deep debt of gratitude to the teachings of this great surgeon on the subject of osteoplastic operation in disorganizing lesions of bone substance, the full value of which, in the past ten years, I have had opportunities to abundantly demonstrate.

The association of a simple fracture with contusions is not an untoward complication, though the frequent necessity of changing the dressings over the peripheral lesions is a painful inconvenience to the patient.

The presence of multiplicity in fracture, comminution, and local, limited disorganization of all the tissues, is always a serious affair. After sustaining an injury of this description, the patient is brought into hospital, to his home, or some temporary abode. At first sight, the limb may seem to be irretrievably lost. It has been twisted on its axis, or is terribly deformed. Torn muscle, tendon and ligament; the bare ends of shattered bones, denuded nerve trunks, with gaping, oozing vessels, everything in contact with the wound is saturated with blood. The limb below is limp, of an ashy white, cold, motionless, and pulseless, all of which tend to produce a most ghastly sight. That it is lost, inevitably lost, is the first impression, and that nothing now remains but the question of its severance from the body—an amputation. Such a summary and hasty conclusion in the past, and not infrequently, we fear, at present, has only too often led to the needless sacrifice of many useful limbs. True, the limb is blanched and cold, and systolic distension of the artery is absent; but more or less shock is present, and, if one take the precaution to examine the sound side, he will find the same physiological phenomena present. But the torn, soiled clothing and particles of dirt and foreign substances mingle with the lacerated tissues, and infection is certain to follow. This latter conclusion is rather assumed than proven.

Now, if we undertake to save the limb,—something which should never be neglected in such serious traumatism in civil life,—unless it is wholly and irrevocably destroyed, the extent and quality of function which we may hope to restore, will, in a very large measure, depend on the nature and the extent of lesion to the bone substance; whether it extends into the articulation; whether there be an equal extent of fracture in both diaphyses; whether there be an extensive shelling out of bone, with complete detachment of the cellular or muscular tissue, loss of integument, muscular tissue or destruction of the arterial trunks.

Before the particular question of the direct treatment of osseous lesions is entered on, it may be well to briefly discuss here other details of importance in all these cases, as anesthesia, hemostasis, antisepsis, or asepsis, and mechanical fixation.

*Anesthesia.*—In the healthy adult, especially when much blood has been lost, it is better, unless our manipulations are to be very tedious, not to use any anesthesia in examination of the wound. The patient may be freely stimulated, by the mouth, with alcoholics before we commence, and, as the raw surfaces are benumbed immediately after injury, little sensation is present until reactory inflammation sets in. Certainly, in the very timid, in young children, we may be compelled to give ether at the time of the first dressing. But with them it is the most dangerous of all. If post-operative collapse and mortal shock are to be avoided, it must be very sparingly administered, and, if possible, by an experienced hand.

The practice of injecting digitalis tincture into the tissues subcutaneously, I hold, is of no value whatever; on the contrary, in an exsanguinated patient it is easy to understand how, through its toxic action, it may cause fatal spasm of the cardiac muscles. It is unnecessary to speak of the possible lethal action of a pulmonary anesthetics in the presence of functional or parenchymatous disease in an organ of the injured.

*Hemostasis.*—As Professor Nicholas Senn lately pointed out in an able brochure, at the late meeting of American Railway Surgeons, modern methods of inducing artificial anemia, or stasis, are not without their dangers to the integrity of the soft parts, when not employed with caution.

There can be scarcely a doubt but in recent times the almost general indiscriminate employment of Esmarch's elastic bandage has contributed very often towards the mortal devitalization and asphyxia of parts which might have been preserved without it. I have seen a case of compound fracture of the leg, attended with a copious hemorrhage, in which a hospital *interne* applied this constrictor for four hours, awaiting the arrival of the visiting surgeon. When the latter came, the foot had hopelessly perished, and had to be amputated. Other similar cases have come to my knowledge. Perhaps, as a prophylactic against hemorrhage this apparatus should be set aside altogether. Tourniquet-pressure over a main blood trunk, with a muslin bandage over the lacerated parts, will serve a useful purpose, temporarily, with more safety than anything else that we have at our command. But even the tourniquet is not to be applied in any case, unless the practitioner be unaccustomed to capital, surgical operations; or in the event of the injury occurring in the night, when good light cannot be had, and assistance is not within reach.

The best suture for vessels of moderate size is none of any kind, but simple effective tortion, as catgut is prone to slip, and silk will irritate. Except in *bleeders* fine gauze-pressure will always easily staunch all peripheral oozing. But should a deep, spouting, retracted vessel continue to well up, it must be fearlessly cut down on and ligated. We must be always on the alert for consecutive hemorrhage in extensive lacerations involving bone.

*Antiseptics or aseptics.*—The application of any sort of chemical solution over clean, non-inflamed, healthy traumatized tissues, is as unnecessary as it is useless and irrational on the molecular, cellular elements of the denuded, freshly opened, compact, cancellous or medullary elements of the bone substance. The efforts of the surgeon should be, rather, to eliminate than introduce all foreign irritants of every description from a healthy wound, particularly when the bone structures are involved.

Nothing can take the place of water, pure and uncontaminated, for both cleansing and hemostatic purposes in this class of cases, but it must not be applied hot on nude disorganized bone. Spring water, when it is not free from an excess of the alkaline salts, is very irritating, and inferior to rain or river water. It is needless to say that water should be about the same temperature as the blood when employed as a cleansing agent, and that by moderately raising or lowering its temperature it is unrivaled as a therapeutic agent in complicated fracture cases.

Asepsis, or cleanliness, a very ancient expedient, though more rigorously employed in modern times, we must rely on rather than antisepsis, in all cases of bone surgery in which there is an entire absence of pollution or wound infection.

On this latter we must "meet steel with steel," and saturate the tissues with a potion, elaborated by the chemist, to neutralize those toxic elements, the products of the microörganisms.

*Mechanical Fixation of the Fragments.*—The ingenuity of surgeons has been fruitful in the elaboration of an infinite variety of mechanical devices for the fixation of bone fragments. There are splints, bandages, weights, swings, supports of various kinds, besides the utilization of spikes, hooks, screws, and wire in maintaining adjustment by being introduced or driven into the bone elements direct. Though this part of our topic ranks only second in importance with hemostasis in osteogenesis, and would richly repay an extended study, yet, in the present instance, restrained by the limitation of time, I must confine my comments on this part of the subject to those apparatuses which are employed in an osteoplasty, and which can be more appropriately considered later on under another head; though I may say, however, here, in a general way, that the simpler and less complicated any fixation appliance is, the better the results; and that, latterly, one of the cardinal and vital elements in the treatment of all mechanical disorganizations of bone shafts, posture has been lost sight of since the introduction of plaster-of-Paris.

### III.

### FRACTURE DISLOCATIONS.

Fracture-dislocations are lesions of a compound character, which, as they involve the arthritic structures, should deserve a special and extended study, will not be dwelt on here only so far as the bone lesion becomes a more or less predominant feature in given cases. Fracture through the elbow and ankle joints, attended with altered relations between the articulations, are familiar examples of this condition, and are remarkable and important injuries ; inasmuch the joint distraint, limitation of motion, and impaired strength after any sort of treatment is, in many cases, occasioned by an excessive or a vicious fusion of osseous hyperplasia, which no one can always either anticipate or obviate after osteogenetic processes are complete.

### IV.

### LOSS OF SUBSTANCE, PARTIAL OR COMPLETE TRAUMATIC AMPUTATION.

Many serious open shattered fractures are attended with loss, or local destruction of parts, the presence and integrity of which are necessary to the process of osteogenesis. It would be absurd, for instance, to think for a moment of saving a limb when the main arterial trunks are destroyed. It would be quite hopeless to attempt to restore a useful limb if both bones are extensively disorganized, with their fragments wholly stripped of periosteum, and detached from it, with no cellular tissue attachments in the adult matured man.

The extensive mistal contusion of a large area of integuments is a serious loss, which may frustrate our best efforts, even when we are quite assured of perfect osseous reconstruction. In a large hospital service, in which it is possible to borrow tissues and fill in the gap by homeoplastic grafting, or transplantation of skin *en bloc*, the difficulty may be overcome. Fiersch's grafts of the scarf skin are practically of no use there. They take promptly, it is true, but there is no wear in them, and they break down on the slightest irritation.

A bone shaft may be saved, but it must have a covering of integument. When there is so much loss of this structure in the hand or foot, that difficulty is encountered in covering a large granulating surface, or when after complete healing there is a great cicatricial contraction, with distortion and immobilization, then the plastic operation of disosossment, or ebonation of a finger, or toe, and the utilization of the member denuded of its bone and tendons, is one of the most valuable procedures known to surgical art; as in extensive disorganizing lesions of the extremities, involving the osseous structures particularly.

### OSTEOGENESIS AND OSTEOPLASTY.

Having considered in epitome the preliminaries indispensable to the successful performance of operations of a conservative character on the osseous structures which have been traumatized, it is now in order to proceed with a consideration of osteogenesis and osteoplasty; or the phenomenon of the growth and regeneration of osseous structures, and how a knowledge of this, with the application of the resources now known to surgery may be utilized in disorganizing injuries which are limited to the extremities.

It is generally well known that the reproduction of bone is influenced by a number of circumstances with which it is well to have an intimate knowledge if we would, with any degree of certainty, forecast results, and give our patients the greatest prospects of recovery with the largest degree of functional restoration.

It is well to remember the influence of age, sex, condition, and treatment. Nor should it be forgotten that while, on the whole, the same common physical and biological elements belong to the entire skeleton; yet, as the various regional bones differ in the functions which they conserve; so do they very widely vary in their properties of repair and regeneration, for there are some bones which cannot be said to ever fuse with their own organic elements after injury, and others still are totally wanting in the way of regenerative power.

For one to read the various and conflicting views of recognized pathologists, with a view of attaining anything like definite knowledge on the precise phenomenon of osteogenesis, is little better than labor lost.

Does the periosteum produce a bone or does it not? If it does, then we must preciously preserve every fragment of it in our dressings. For centuries this question has been a matter of dispute, and it is so yet, notwithstanding the revelations of the microscope. For, at the present day, even so eminent an authority as is Dr. McEwen, of Glasgow, emphatically denies that the periosteal envelope possesses any regenerative power at all. Some have gone into "hair-splitting" differences, denying that as a membrane it can regenerate bone, but that its inner layer, or lamina, is a powerful regenerator of osteoplastic cells.

While my own observations on a large number of open osseous lesions convince me that anything approaching perfect regeneration is impossible in the absence of the periosteum ; still there can be little doubt that the medullary membrane is an important auxiliary in yielding bone cells, as well as other tissues in the immediate vicinity of the injury. It has long been known, too, that through molecular transmutation, blood-clot may part with its aqueous elements and undergo nucleation, segmentation, fibrillous, cartilaginous, or osseous changes. In those fractures extending into the epiphyseal or apophyseal ends of shafts, it is fair to assume, from analogical physiological phenomena, that bone substance may be wholly regenerated by endochoroidal formation.

Many authors, in describing fracture, so restrict themselves to the bone injury alone that the casual student might assume that this alone is what should command one's sole attention, but this is a short-sighted, delusive notion. All fractures, with very rare exceptions, are, strictly speaking, compound, though in surgical nomenclature none are so designated, except such as are in direct contact with the atmosphere. This opening of the tissues and exposure of the denuded ends of the bones is always an unfortunate complication. Under the most intelligent surveillance and environment, it greatly retards processes of repair and consolidation. When one of those compound fractures extends into a joint, or involves a major shaft, as the humerus or femur, the degree of inflammatory reaction is severe, often resulting in fatty degeneration of the compact substance, anchylosis, or even loss of life. Since the advent of the antiseptic doctrine, not a few imbued with a conviction that the employment of chemical solutions secured them immunity, have many times come to grief, crippled or lost their patients, when they imprudently, by the free use of the scalpel, made a simple a compound fracture, either for purposes of diagnosis, or direct mechanical treatment.

But why such disastrous consequences to open fractures when lethal elements of the atmosphere may be excluded. If we carefully observe the physiological properties of bone, we will readily comprehend the *raison d' étre.*

What most strikingly impresses us at the outset is the extreme intolerance to irritation of exposed disorganized bone wherever situated, although this is more marked in certain bones than in others, and in different sections of the same bone. This is most obvious, however, in the tubular segment of the shafts.

Condition and position exert a marked influence in the regenerative processes after fracture. For instance, as a rule, in fracture, the sooner, after all turgescence and tumefaction have disappeared, that adjustment and rest is given the fragments, the more prompt is union. Yet, in many cases of fracture of the lower extremities, the completion of union is often rather accelerated by slight motion in the joints above and below the point of fracture, and by carrying the limb in the vertical position. Moderate motion, then, is not incompatible with union, but in many it favors osteogenesis, as well as the rapid absorption of adventitious elements. It also very materially aids in the prevention of anchylosis, a condition so prone to follow the protracted fixation of an articulation. Sampson Gamgee[1] was among the first to call attention to the fact that the union of bone after fracture is effected in a large measure, as the union of the soft parts, viz., by primary and secondary fusion of the fragments by a plastic, non-inflammatory hyperplasia ; and by the more tedious and imperfect process of large effusion, condensation, and ossification, which is akin to the common granulation of wounds.

Gamgee taught that when there is a simple, uncomplicated fracture, without much displacement, and its immediate reposition is possible without protracted splint pressure, in the healthy and vigorous, *immediate union*, or perfect union, within a few days or a week, occurred. My own experience goes to support this statement, a knowledge of which cannot be overestimated in the therapy of fracture. And in those cases in which a corporation surgeon, finding no crepitus, displacement, or callus after a few days or a week, might hastily assume that there had been no fracture, and assume that extortion was being practised.

### OSTEOPLASTY.

Ollier[2] divides osteoplastic operations into three classes, which, for their utility and simplicity of arrangement, will be adopted here : (1.) Autoplastic. (2.) Homeoplastic. (3.) Heteroplastic.

He ranks autoplasty, or the transference of grafts, or transplantation of osseous tissue, from one region or position in the same individual, as that which is the most successful.

1. Sampson Gamgee. Clinical Surgery. Page 217.
2. Traité Experimentale et Clinique de la Régénération Dis Os.

Homeoplasty, or transplantation of living bone from one individual to another, or one animal to another of the same species, comes next in importance.

He places heteroplasty, or the implantation into the human economy of the bone elements from the lower animal, as the least useful, and finally questions whether it possesses any utility at all.

### AUTOPLASTY.

The field of autoplasty is very large, and with the daily increase of traumatisms, which involve the extremities, it is constantly attaining greater proportions. However, certain definite conditions must be observed to make its application practicable, or possible, in any case. The injury to the bone must be recent, before inflammatory changes are manifest. The fragments must have an ample nutrient supply, and be protected from infection or irritation.

We may have a comminution of bone in simple fracture, which Dr. Charles Powers designates the "bean-bag" fracture, which gives rise to no inconvenience, all the particles consolidating in the process of cure. But when we have to deal with an open, compound fracture attended with much shattering, then every fragment must come away at the primary dressing, which is completely detached and lying loose in the wound, whether it has a periosteal investment or not.

I am well aware that this is not in keeping with the tenets of surgery, as expounded by many modern authors. But we must not disregard the evidence of our own senses, nor the accumulated observations of many others, who affirm that all fragments completely cut off from a direct vascular supply must sooner or later perish in a fracture. It has been thought that we might retain or replace such fragments, and that they may become ossific centers. In my own experience, when this has been attempted, the imprisoned particles have either become encapsulated, underwent a gradual absorption, or were thrown off by a process of ulceration. Nevertheless, it must be acknowledged that autoplastic bone grafts, as skin grafts in torpid, languid ulcers on the surface, though they melt down and are taken up by the absorbents, or undergo necrotic changes, they do in some way, in may cases, exercise a marked stimulative effect on regenerative processes.

With those fragments of bone which preserve a vascular connection with the connective tissues, through the periosteum or their endochondrium, re-implantation is immediately successful and definite in its results.

### CLASS OF CASES IN WHICH AUTOPLASTY OF BONE MAY BE UTILIZED WITH THE GREATEST ADVANTAGE, AND THE PRECAUTIONS NECESSARY TO OBSERVE TO SECURE THE MOST EFFICIENT RESULTS.

Osteoplastic procedures are especially valuable in a large number of compound fractures in the bones below the knee- or elbow-joints, attended with more or less shattering and displacement.

When the fracture is single, and the fragments are not stripped of periosteum, complete reduction of the fragments, with appropriate dressings, leads to rapid ossification, obliteration of the hiatus, and solid union. Unfortunately, complete fixation of the fragments by any sort of mechanical adjustment which will retain the broken ends of the bones, without inducing dangerous pressure in the main blood-trunks, and imperiling the soft parts, is only too often utterly impossible.

It was hoped, with the protection which antiseptics promised, that this difficulty would be obviated by extending mechanics to the bones direct; that metallic spikes, or sutures, might be utilized in splicing the fragments, removing these materials after an osseous shell of new bone was formed, or fusion of the ends commenced. In this particular we have been doomed to disappointment, for, with very few exceptions in those cases, none of these expedients are of any value whatever ; nay, on the contrary, the introduction of wire sutures to maintain an efficient grip on the ends of the bone, entails such a violent manipulation of it by the heavy forceps and drill that it seems to rather retard than accelerate union. Wire corrodes at points where it impinges on the bone; on its medullary and periosteal surfaces necrosis occurs, and little progress is made until it is removed. But in rare special cases, these materials, notwithstanding their drawbacks, may be employed with some advantage. By properly employing posture, reducing or preventing muscular spasm, we may, in the majority of cases of single compound fracture of the forearm or leg, secure union without any more deformity or shortening than had there been an *unopened* fracture. When this follows, the result must be considered as very good.

In compound, multiple fractures, in which there are more than one fracture of either or both bones of the leg, with mutilation of the soft parts, and considerable displacement of osseous substance, the total sacrifice of the limb or the degree of its future usefulness will mainly depend on the efficiency of osteoplastic intervention.

In the growing youth, or adolescent, a shaft may be totally disorganized from one epiphyseal line to the other, being completely shelled out of the periosteum, when this membrane is amply competent to fully restore the lost bone substance. Such an example has come into my hands, which, at first sight, seemed hopelessly lost, but the patient now, six years after the accident, has a strong and useful limb, and walks without a limp.

In double fracture of a shaft, much may be accomplished in the child or adult in the way of structural regeneration, but when the fracture is triple or quadruple, continuous and complete ossification of the bone segments is rarely possible, though if each preserves its periosteal investment, and is on any of its surfaces firmly united to the cellular-vascular structures, it will serve as the nidus of a new ossific center, which, after a slow process of exfoliation, will throw off the devitalized, necrosed bone blocks, and substitute in their stead a mass of amorphous bone elements ; the cement substance of repair being supplied by a series of concentric layers, advancing in a centripetal direction until the border of each meet, and the continuity of the diaphysis is again restored. In this connection, it may be well to have it distinctly remembered, that every species of osteoplasty, the simplest as well as the most complicated, though Nature's best effort at regeneration, is but a species of mending or patching, analogous with and identical to the repair of the integuments or cellular tissues ; and that true, perfect structural bone is never reproduced, its prototype, or substitute, in composition and function, however, bearing a close resemblance to it.

The conditions, then, necessary for successful autoplastic operations on traumatized bone, are, first, youth and sound health ; second, the adhesion of the fragments and periosteum to the cellular tissues ; and, third, a moderate extent of bone lesion. When both shafts are equally damaged in their continuity, and the adjacent neural and vascular elements have sustained great violence, efficient osseous restoration is seldom realized.

In serious fractures of the forearm, as muscular relaxation in this situation is easily secured, and perfect fixation of the fragments, with ample bodily exercise, are not impracticable as in the lower extremity, osteoplasty should be resorted to with great diligence, as the prospects of success are greater here than in any other region of the body.

With the humerus and femur, directly the opposite condition obtains. If efficient osteoplasty were a safe expedient, how great would be the temptation in every case of fractured femur to cut down, make an accurate diagnosis, bring the displaced fragments of bone into *direct* contact, and fix them there with the spike or suture?

Then the inevitable shortening of the femur of the adult in fracture would be obviated, and perfection in function would be restored. In bad fractures of the humerus, deformities or diminished action would be replaced by symmetry of action and former strength. But the medullary membrane of these shafts is, on mechanical manipulation, extremely susceptible to a dangerous phase of inflammation, which is prone to excite such a general pyemic state as to lead to amputation of the limb. This is almost the invariable result when the femoral shaft, in simple fractures, is rendered compound, and the ends of the fragments are pegged or wired. This same danger is always present, though not in the same degree, in fractures of the humerus.

Direct osteoplastic procedures instituted with a view of securing adjustment and immediate union in simple fractures, should be condemned as impracticable, endangering the integrity of the limb, and often imperiling life.

The re-implantation of bone, or an autoplastic operation, in a comminuted fracture with a free opening, is a valuable procedure when the fragments, a few or many of them, maintain a connection with the soft parts, and preserve a part of their periosteal covering. It must be confessed, however, that even here the detached fragments are gradually thrown off by the new crop of bone cells, which spread in every direction during the process of bone regeneration. But we will encounter cases in which the bone fragments lie loosely in the mangled tissues, which have no investment of any description, and which, on their removal, leave an enormous breach. What shall be done here ? The bone elements are, possibly, yet vascular and uncontaminated ; and it would seem that by their replacement and fixation each fragment would become a new ossific center. With them, nevertheless, ulceration and necrosis is the rule, the devitalized bone acting as a foreign substance until it is thrown off.

In very many instances, when these serious complicated fractures come under our observation, it may be quite impossible to institute a sufficiently searching examination to determine, with certainty, the vitality of the fragments, but there is nothing to be lost by delay; by waiting from twenty-four to forty-eight hours before we definitely decide what, if anything, can be accomplished by osteoplasty, when Nature will map off the devitalized from the living, and we may estimate with a considerable degree of accuracy what the ultimate outlook will be.

Certainly, in leg fractures, when the fibula has escaped, or there is but one fracture of this deeply buried, brittle shaft, it serves, in a large measure, as a splint, and aids greatly in fixing the limb. When this bone shares in the shattering, the chances of osseous restoration are greatly diminished, because the bone is so deeply lodged, and is so fragile, possessing so little medullary and cancellous substance, that little can be done with it when extensively shattered, and its loose fragments lie in the open wound.

### HOMEOPLASTIC OPERATIONS.

Homeoplasty, as its designation implies, is the transplantation of bone from one part of an individual to another, or from another of the same species. I have never had any experience with this phase of osteoplasty, nor can I conceive of it possessing anything other than a very limited range of application.

Its efficiency, however, is vouched for by Ollier, Horsley, McEwen, and others in certain rare cases. When it is remembered that it is only when the bone preserves its attachment with the cellular elements that autoplasty is feasible, it is obvious that unless we can coaptate the fragments of two different individuals by immediate segmentation of the fragments until they have become fixed, as we do in skin grafting, we must fail of success. But physiological laws and the moral sense are both alike antagonistic to such mutilating experiments.

### HETERO-OSTEOPLASTY.

Of late years, so many heterodoxical ideas in connection with osteogenesis have been promulgated and taught, that it commenced to seem, for a time, that our old masters were all wrong in the views which they held as late as twenty years ago on the subject of bone regeneration. Hence, we must learn anew the principles of bone surgery.

Far-reaching and of wide proportions were the new vistas opened to us. It was said that not only could bone be completely removed from all its connections for varying periods of time, and then be restored with impunity, but that we might blend the osseous structures of the lower animal with man. It was alleged that antiseptics opened the way to all this. The periosteum of the kid, the young rabbit, and the guinea-pig, as well as their soft cartilaginous shafts, were utilized as ossifying centers. The vitelline membrane of the hen's egg and the medullary membrane of the chick's femora were also appropriated for like purposes.

It was even alleged that we might revitalize dead bone by placing it in contact with the living, as in ossifying bone cavities with particles of decalcified bone. The human being and the lower animal have been locked together with a view of borrowing from one to supply a bony defect in the other.

Notwithstanding all that has been said and written of late on this subject, it may be safe to affirm that the grounds occupied by physiologists and surgeons in the beginning of the present century on osseous regeneration are yet quite impregnable, and that, taken as a whole, we may assert of the most modern views on hetero-osteoplasty, that there is nothing in them, and that it is as well to totally ignore them.

It won't do to tell us that because certain bones seem to ossify more rapidly after the introduction of ossific substances, that they imparted certain stimulating properties, and assimilated with living bone. Many of such cases have been prematurely reported, when later the whole mass has been absorbed, or worked its way through to the surface by ulceration.

Hetero-osteoplasty, then, is not an operation which time and an abundant experimentation justify as applicable to traumatized bone disorganization in the extremities of the human subject.

In those cases of double fracture of one shaft as the tibia, with a single fracture of the fibula, if the central segment of the tibia is shelled out in one or many fragments, what shall be done?

(1.) Shall we be content with bringing the limb into position, adjusting the fibula, allowing the chasm left by the removed fragment, the tibia to remain, with the hope that by periosteal regeneration it will be filled in? (2.) Will we resort to homeoplastic or heteroplastic methods of bone grafting. (3.) Will we, on the contrary, cut down on the fibula, and remove enough of its shaft to permit the separated ends of the tibia to come together and so unite? (4.) Or, lastly, will we permit the parts to unite, leaving the gap between the ends of the tibial fragments to heal, hoping that by compensatory hypertrophy, and prothetical appliances, to be able to secure a fair degree or restoration of function in the leg and ankle ?

The first may succeed in the youth under twenty, while the growing skeleton is in process of growth, though even then it may fail. If the disconnected bone can be rendered aseptic with this class, it may be replaced, serving, mechanically, only as a splint, and being a substance least likely to excite irritation, until an involucrum of new bone entombs it.

The first question has been answered under the head of autoplasty.

The second is answered under homeoplasty, autoplasty, and heteroplasty. The former, as a conservative measure, may avail, but in the latter failure will almost inevitably result. As to the third and fourth propositions, in compound fracture of the leg I have found it a measure of great utility in the healthy. When the loss of tibial bone substance is limited in the adult, we may with safety and with advantage cut down on and remove a sufficient segment of the fibula to permit the divided and separated ends of the tibia to come together. But it must be done *immediately* after the injury if we would secure solid union. As to the fourth question, if we have doubt as to our patient's recuperative energy, and would avoid additional shock, we must be satisfied with leaving a gap and cushion joint, the resulting weakness in the limb being compensated for by a mechanical adjustment. The latter in many ways, when a choice is open to us, is the preferable.

With a loss of more than a third of the tibial shaft, especially when this is limited to the upper segment, though the entire fibula remain and more or less compensatory hypertrophy follow, yet through loss of muscular attachment resulting, want of support and steadiness at the ankle-joint, will render the limb very defective in function, so that the question of amputation and the adjustment of an artificial limb must then be considered.

It goes without saying, that in extensive compound fracture of the bones of the forearm in the young, in whom the bones are elastic, succulent, and immature, with a thick periosteal sheathing, very much may be accomplished by osteoplastic methods.

As these bones perform a complexity of function, in force and prehension, it is evident that perfect restoration in action need not be expected after any osteoplastic operation on them. Perhaps, after the healing processes have been completed a flail joint may result, or so much of the shaft of one of the bones may require resection, as to require thereafter a radial ulnar support. If the carpal end of the radius or the humeral end of the ulna is so extensively involved as to require its entire sacrifice, though the remaining structures of the limb preserve their integrity, muscular power is so much diminished, control of joint action lost, and an edematous state of the tissues follow, that the hand serves no useful purpose is a source of constant discomfort, and a useless, burdensome appendage.

I am unacquainted with any case on record of epiphyseal, or apophyseal regeneration after loss by physical violence. Hence, in those rare cases in which the entire osseous composition of a joint is by force swept away, yet life is spared, its restoration need not be anticipated, as nothing but a pseudo-arthroses is provided by Nature as a substitute for the normal.

As this essay has been written with a view of considering in outline only a few general features in connection with the interesting question of osteogenesis and osteoplasty in those disorganizations of bone substance resulting from force or violence however applied, my efforts under the circumstances might be regarded as incomplete if no specific reference were made to those lesions of bone consisting of fracture, shattering, or crunching of the osseous structures, occasioned by railroad violence, on the steam railroad, in contradistinction to the tramway.

It cannot be emphasized too strongly, that those railroad injuries which involve the soft parts and encroach on the bones always possess attributes peculiar to themselves.

The distinguishing characteristics attending serious injury of the extremities by railroad rolling stock are, first, the serious and profound constitutional disturbance, which is so constantly associated with this class of traumatisms. These are of a mental and physical character. The first is confined to and expressed through the sensorium; the second are of a general and local description.

In order to essay any sort of osteoplastic procedure on the living body, we must be quite assured that our patient will rally from primary shock.

Now, in those dreadful railroad crushes, in that one or two second interval between the full realization of the dreadful calamity, which there is no escape from, the instant of contact, a flash, of horror suspends all the vital functions, the cerebral and spinal centers are palsied, and consciousness of the external world is lost. The heart stops, or beats with a feeble, irregular stroke. But in a moment all is over, the mangled man lies momentarily dead. However, reaction soon sets in, when the injury is not of a mortal character. But shock, though temporarily overcome, will make an indelible impression on our patient and the nutrition of his wound. And, perhaps, at a time when we hope all immediate danger is past, bulbar paralysis may set in, and our patient sink.

It is seldom when a limb is badly crunched by car wheels, that the body does not sustain a violent concussive twist, or jerk, is violently dragged or crushed, and the internal organs, as the spinal cord, liver, kidneys, or lungs suffer in varying degrees. It is well known that after all violent railroad accidents, the renal secretion is markedly affected, *diabetes*,—insipidus, or melitus, temporary or chronic,—often supervenes.

Hence, here the condition of the general system or various complications demand our most careful consideration, else our best endeavors at the seat of injury will be futile, as distant pathological conditions may lead to a fatal termination, when ossific processes are most promising.

### DISTINCTIVE PECULIARITIES OF THE LOCAL LESIONS IN RAILROAD MUTILATIONS.

In such pathological conditions as are met with in practice which require an amputation in continuity, the operator may map out his flap-areas with a mathematical accuracy, and secure prompt primary union. When a limb has been fractured by the body having been propelled upon it, as it is in a fall or in a projected movement, or when a limb has been run over by any ordinary street vehicle, we may, with a considerable degree of certainty, when a traumatic amputation has been effected, resect at a vital line through the proximal tissues without fear of subsequent gangrene.

In railroad crushes of an extremity, none of these conditions obtain, for the magnitude of force and momentum are commonly so great that the tissues are destroyed in both directions far beyond the point of contact, some to a greater degree than others; and, immediately after injury, there is no means known to science to enable us to limit the precise extent of destruction. The vessels and muscles bear the consequence of damage the best, the integument and bone being the first to succumb. The bone substance, which at first seems vascular, soon parts with its vermillion hue, looses its firmness, and breaks down through necrotic processes, or by a molecular, interstitial osteomyelitis, so that often, in a short time, they proceed far beyond the point of fracture before sound, healthy bone is formed.

Again, with not a few, though osteogenetic processes are active and bone restoration is promising, but, alas, the broad plaque of skin which we depended on to enclose the stump is occupied by active gangrenous changes, thereby rendering all our best conservative efforts useless.

The above description, which, it will be agreed, is not an overdrawn one, should not be lost on those whose province is and always should be directed towards saving life and limb. It teaches us that in railroad disorganizations, unless the damage to the parts has been so great as to produce a traumatic amputation, we must limit our interference at the primary dressing to cleansing the wound, subduing all hemorrhage, bandaging and bracing the mangled limb, and waiting until time and natural physiological phenomena have clearly determined whether the limb must be sacrificed, or that the part may yet preserve sufficient vitality to permit us to so successfully utilize modern osteoplasty so as to spare to the unfortunate patient a useful and serviceable member. This later will be all the more gratifying to the patient, and an immense relief to the corporation in whose employ we may serve, or the injured was employed. In civil life, with the class of injuries here described, *delay* should be the watchword, as in our times, under proper circumstances, very much may be gained, and many limbs spared by observing this course, while prompt, immediate primary amputation, a procedure always full of peril, destroys at once and forever every prospect of restoration.

The subject which I have essayed to treat of is one of such prodigious proportions and enormous importance, that however fascinating its study is to us all, as I am limited by time, I must ask your indulgence if it has been very incomplete and wanting in such a classification or subdivision as might enhance its value.

[NOTE.—The limit of this brochure will not permit of considering, in detail, either traumatic amputation, or compound fracture dislocation, according to classification.]

302 W. FIFTY-THIRD STREET.

